# Pulsed-Field Ablation Is Associated with Lower Endothelial Injury and Procedure Time Compared to Cryoballoon Ablation in Paroxysmal Atrial Fibrillation

**DOI:** 10.3390/pathophysiology32040060

**Published:** 2025-11-07

**Authors:** Josip Katic, Ante Anic, Toni Breskovic, Josip Andelo Borovac, Branka Kresic, Daniela Supe-Domic, Marko Kumric, Josko Bozic, Zrinka Jurisic

**Affiliations:** 1 Cardiovascular Diseases Department, University Hospital of Split (KBC Split), 21000 Split, Croatia; josipkati@gmail.com (J.K.); anteanic@gmail.com (A.A.); toni.breskovic@gmail.com (T.B.); zrinkacn@gmail.com (Z.J.); 2 Department of Health Studies, University of Split, 21000 Split, Croatia; brkresic@gmail.com (B.K.); daniela.supedomic@gmail.com (D.S.-D.); 3 Department of Pathophysiology, University of Split School of Medicine, 21000 Split, Croatia; marko.kumric@mefst.hr (M.K.); josko.bozic@mefst.hr (J.B.); 4 Department of Medical Laboratory Diagnostics, University Hospital of Split (KBC Split), 21000 Split, Croatia; 5 Department of Internal Medicine, University of Split School of Medicine, 21000 Split, Croatia

**Keywords:** atrial fibrillation, pulmonary vein isolation, pulsed-field ablation, cryoballoon ablation, endothelium, von Willebrand factor, thromboembolism

## Abstract

Background: Thromboembolic events, though infrequent, remain a significant complication of atrial fibrillation (AF) ablation, largely related to endothelial damage. Cryoballoon (CB) and radiofrequency ablation can induce pro-coagulant responses, whereas pulsed-field ablation (PFA), a novel non-thermal electroporation-based technique, has shown tissue selectivity with potential endothelial-sparing effects. Methods: We aimed to compare PFA and second-generation CB ablation regarding endothelial injury in patients with paroxysmal AF. In this single-center prospective observational study, 25 patients with paroxysmal drug-refractory AF underwent pulmonary vein isolation using either a pentaspline PFA catheter (n = 14) or a second-generation CB catheter (n = 11). Circulating von Willebrand factor antigen (vWF) levels were assessed before and after ablation as a biomarker of endothelial damage, alongside routine laboratory and echocardiographic parameters. Procedural characteristics were also analyzed. Results: Baseline demographic, clinical, and echocardiographic data were comparable between groups. PFA was associated with significantly shorter skin-to-skin procedure time (59 vs. 94 min, *p* = 0.005) and left atrial dwell time (44 vs. 79 min, *p* < 0.001) compared with CB ablation. Importantly, vWF levels decreased significantly after PFA (−7.6%, *p* = 0.007), while CB ablation showed a non-significant increase (+9.5%, *p* = 0.155). The between-group difference in percent change of vWF was statistically significant (−5.6% vs. +8.3%, *p* = 0.006). Conclusions: PFA was associated with reduced endothelial injury and shorter procedural times compared with CB ablation, suggesting a potential advantage in lowering thromboembolic risk. These findings support the concept of PFA as an “endothelial sparing” ablation modality. However, the PFA procedure was associated with a significantly greater extent of myocardial injury, as reflected in circulating high-sensitivity cardiac troponin T values, compared to CB ablation (*p* = 0.007). Larger, randomized studies are warranted to confirm these results and evaluate long-term clinical outcomes.

## 1. Introduction

Thromboembolic complications, although rare, remain one of the most relevant adverse events of atrial fibrillation (AF) ablation and are closely linked to endothelial damage [1,2]. Traditional energy sources such as radiofrequency (RF) and cryoballoon (CB) ablation can induce a procoagulant response, with elevated tissue plasminogen activator and von Willebrand factor (vWF) levels, predisposing patients to thrombosis and stroke [3]. Pulsed-field ablation (PFA) is a novel non-thermal technique that creates lesions through irreversible electroporation using ultra-short electrical impulses [4]. Preclinical and translational studies demonstrated that PFA is highly tissue-selective, effectively ablating cardiac myocytes while largely preserving the endothelium and extracellular matrix [5,6]. This endothelial-sparing effect may translate into a lower thromboembolic risk compared with thermal ablation modalities. To date, however, no clinical study has directly compared PFA with CB ablation regarding the extent of endothelial injury. Therefore, this study was designed to address this knowledge gap by assessing biomarkers of endothelial damage after pulmonary vein isolation (PVI) with PFA versus CB.

## 2. Materials and Methods

This prospective observational study included 25 patients with paroxysmal drug-refractory AF in whom the biomarkers were evaluated during the peri-procedural period of the PVI using a pentaspline PFA catheter (Farawave, Farapulse Inc.; Menlo Park, CA, USA) or 2nd-generation CB catheter (POLARx, Boston Scientific Inc., Marlborough, MA, USA). The study was conducted at the Cardiovascular Diseases Department, University Hospital of Split, Croatia. The strategy was decided before the procedure started at the physician’s discretion.

AF was classified as paroxysmal if episodes spontaneously converted to sinus rhythm within seven days or were terminated by electrical or pharmaceutical cardioversion within 48 h [7]. Exclusion criteria included age under 18, non-paroxysmal atrial fibrillation, prior PVI, additional left atrium substrate modification, severe structural cardiac abnormalities, active thromboembolic process, renal or liver failure, coagulopathy, thrombocytopenia, known malignancy, and recent surgical procedure. The study protocol was approved by the Ethics Committee of the University Hospital of Split (Class: 500-03/21-01/100, Number: 2181-147/01/06/M.S.-21-01), approval date 21 January 2021. The study adhered to the ethical guidelines in the Declaration of Helsinki as reflected in a priori approval by the institution’s Ethics Committee.

For each patient, a comprehensive medical history was undertaken. Patients had a physical examination, 12-lead electrocardiogram (ECG) recording, and baseline anthropometric measurements. All patients were treated according to current evidence-based medicine and guidelines. Transthoracic echocardiography was done using the Vivid IQ platform (GE HealthCare, Chicago, IL, USA) for every enrolled patient. The same experienced echocardiographer, blinded to the treatment assigned, obtained all echocardiographic measurements according to the European Association of Cardiovascular Imaging recommendations for cardiac chamber quantification [8]. Simpson’s 2D bi-plane approach assessed left ventricular ejection fraction (LVEF). The left atrial diameter was measured in the parasternal long-axis view.

Fasting blood samples for determination of D-dimer, brain natriuretic peptide (NT-proBNP), high-sensitivity cardiac troponin T (hs-cTnT), and other routine biochemical parameters were taken for all participants one day before and one day after the procedure. All samples were analyzed in the same laboratory by an operator blinded to the subject’s assignment in the study group by good laboratory practice. Von Willebrand antigen (vWF Ag, Siemens Healthineers, Erlangen, Germany) was used to quantify the degree of endothelial damage. Blood samples for vWF Ag were collected from the left atrium at two pre-specified time points during the procedure: before ablation (after heparin administration and transseptal puncture) and after the last PV isolation. Before collecting the sample, the first 10 mL of blood was discarded. The samples were immediately centrifuged, and plasma was aliquoted and stored at −20 °C until analysis. Samples for vWF Ag were analyzed on Atellica COAG 360 System coagulation analyzer (Siemens Healthineers, Erlangen, Germany). The high-sensitivity cardiac troponin T (hs-cTnT) and N-terminal (1–76) pro-brain natriuretic peptide (NT-proBNP) concentrations were determined using the electrochemiluminescence (ECLIA) method using the Eclesys^®^Cobas e801 Roche Diagnostics (Roche, Manheim, Germany). Standard flow-cytometry-based hematologic analyses (ADVIA 2120i, Siemens Healthcare, Erlangen, Germany) were utilized to ascertain the complete blood count. Standard laboratory methods (Cobasc702, Roche Diagnostics, Manheim, Germany) were used to measure the concentrations of glucose and creatinine in fasting plasma. D-dimer was determined by using standard analyses (BCS, Siemens Healthcare, Erlangen, Germany).

The ablations were performed under conscious sedation with a bolus of midazolam and fentanyl, followed by a continuous propofol infusion. Unfractionated heparin boluses (150–200 IU/kg each), depending on previous oral anticoagulants, were administered. Activated clotting time (ACT) was monitored at 30 min intervals, followed by additional heparin boluses to maintain an ACT of 350 s. Before ablation, the presence of a left atrial thrombus was excluded by using either pre-procedure transesophageal echocardiography or intracardiac echocardiography (ICE) (AcuNav system, Siemens, Munich, Germany). In the following cases, we used our standard approach to perform PVI with the PFA technique. After a single transseptal puncture, the Farawave ablation catheter was advanced to the ostia of all PVs: left superior PV, left inferior PV, right inferior PV, and right superior PV. ICE imaging and fluoroscopy were used to monitor catheter positioning at the antral level. A train of five consecutive waveforms was delivered for each application, accounting for a total of 2.5 s of ablation time. We used two catheter configurations in each PV ostium: four applications in the ‘flower’ configuration, with the catheter shape fully open, and four applications in the ‘basket’ configuration, with the catheter shape partially closed. The Cryoablation was performed with a single transseptal puncture, and a steerable 15-Fr sheath (POLARSHEATHTM, Boston Scientific Corporation, Marlborough, MA, USA) was introduced into the left atrium. Then, by moving the balloon catheter over an inner lumen circular mapping catheter, a 28 mm CB (POLARxTM, Boston Scientific Corporation, Marlborough, MA, USA) was advanced toward each PV ostium (POLARMAP^TM^, Boston Scientific Corporation, Marlborough, MA, USA). Before ablation, PV potentials were recorded at a PV ostium using a mapping catheter (POLARMAP^TM^, Boston Scientific Corporation, Marlborough, MA, USA). Following balloon inflation, operators repositioned the catheter under fluoroscopic guidance to seal the PV ostium completely. Before ablation, complete PV occlusion was documented using selective contrast injection. If the time to isolation (TTI) was 60 s or less, cryoablation applications lasted 180 s. However, if the TTI exceeded 60 s, a cryoablation application of 240 s was carried out. If no PVI was obtained, additional freeze applications were carried out. Bi-directional PVI in sinus rhythm was confirmed after PV cryoablation at a proximal site in the PV ostium. To detect phrenic nerve palsy during right PVs ablations, right phrenic nerve pacing with a deflectable quadripolar catheter was performed. Skin-to-skin procedure time is defined as the time from the first sheath inserted to the last sheath removed from the patient. In all cases, pericardial effusion was excluded with ICE after the procedure, and the access site was closed with a ‘modified figure 8’ suture.

All data analyses were performed using SPSS Statistics for Mac^®^ (version 25.0, IBM, Armonk, NY, USA) and Prism 6 for Mac^®^ (version 6.01, GraphPad, La Jolla, CA, USA). Data were presented as mean ± standard deviation (SD) or median (interquartile range) based on the variable distribution normality or number (N) with percentage (%) within the particular category of interest. The normality of distribution for continuous variables was assessed with the Kolmogorov–Smirnov test. For differences between groups of interest (PFA vs. CB), an independent samples *t*-test was used for continuous variables with normal distribution, while the Mann–Whitney U test was used for continuous variables with non-normal distribution. The chi-squared (χ^2^) test determined differences between groups regarding categorical variables. For the main analysis, circulating levels of vWF were examined in the “before–after” analysis in which the initial values (one day before the ablation procedure) of each patient were pairwise compared to the values obtained after the ablation procedure (one day after the procedure). The mean percent change from before to after the ablation procedure was calculated for each group using the following formula: [(Measurement2 − Measurement1)/Measurement1] × 100, and these values were then compared between both groups of interest by using an independent samples *t*-test. All results that reached a significance level (*p*) < 0.05 were considered statistically significant, and two-tailed *p*-values were reported in all instances.

## 3. Results

Among 25 consecutive patients with paroxysmal AF, 14 patients underwent PFA and 11 underwent cryoablation, respectively. The mean age was 61 ± 10 years, and 56% were female. Arterial hypertension was present in more than half of the patients (52%), while one had diabetes mellitus (4%). The mean LVEF and LA diameters were 64 ± 5% and 42 ± 5 mm, with no difference between the two groups. The median duration of AF since the initially recorded episode was 60 months (IQR 36–126). The baseline, clinical, echocardiographic, and laboratory data did not significantly differ between the two examined groups, as shown in Table 1.

Furthermore, there was a considerable decrease in the skin-to-skin procedure time and left atrial dwell time in the PFA group compared to the cryoablation group, as shown in Table 2.

**Figure 1:**
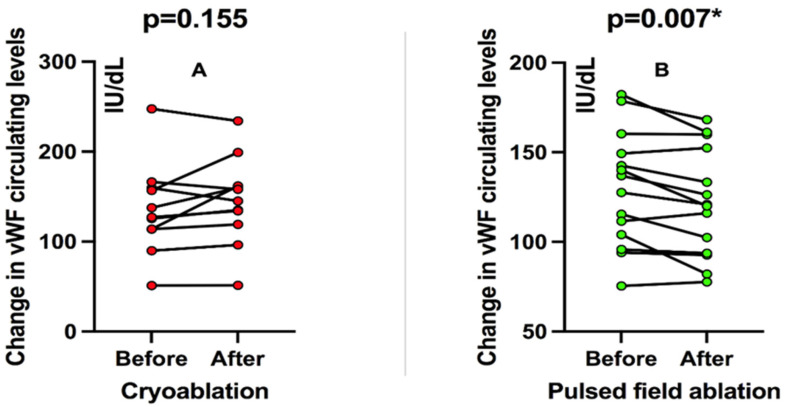
(**A**) Change in vWF circulating levels before and after cryoablation. (**B**) Change in vWF circulating levels before and after pulsed-field ablation. vWF: von Willebrand factor. * Significant result with *p* < 0.05.

In patients treated with cryoablation, there was no significant change in circulating vWF levels before and after the ablation procedure when patients were analyzed pairwise in a “before-after” fashion (an increase of 9.5%, *p* = 0.155; Figure 1A). On the other hand, patients treated with PFA showed a significant decrease in circulating vWF levels compared to before and after ablation (decrease of 7.6%, *p* = 0.007; Figure 1B).

Finally, when both groups of interest were analyzed in terms of the mean difference in percent change (%) of circulating vWF before and after the ablation procedure, in a non-pairwise fashion, it could be observed that patients receiving PFA, on average, had a decrease of 5.6 ± 7% while patients receiving cryoablation had an 8.3 ± 15% increase in the circulating vWF levels.

**Figure 2:**
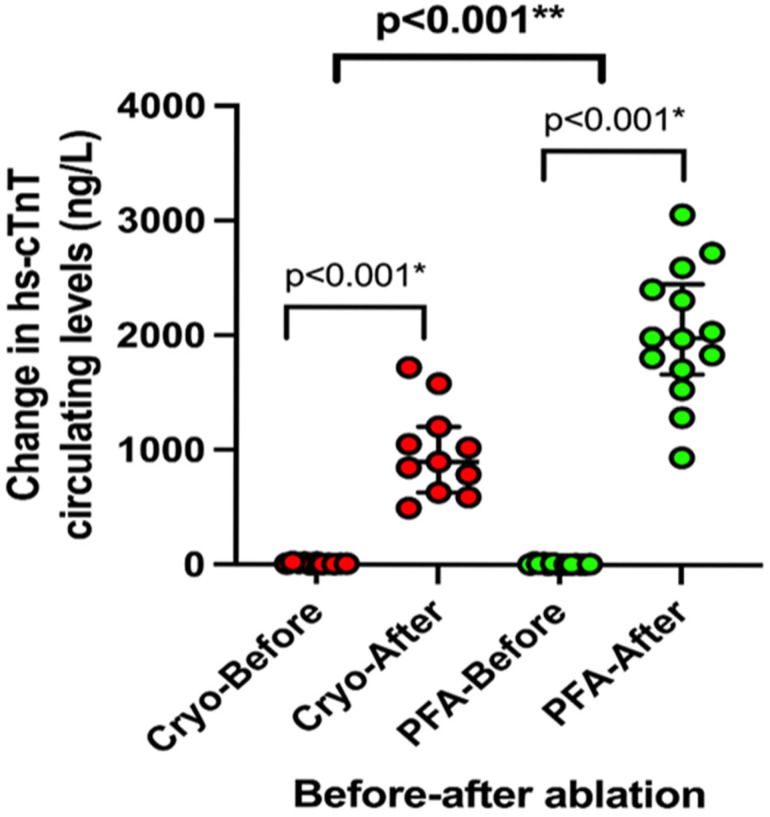
* Change in hs-cTnT circulating levels before and after cryoablation and pulsed-field ablation. ** The difference between cryoablation and PFA. hs-cTnT: high-sensitivity cardiac troponin T; Cryo: cryoablation; PFA: pulsed-field ablation.

Compared to baseline, the median post-ablation hs-cTnT values were significantly higher in the PFA group (hs-cTnTpre 6.0 (4–13) ng/L vs. hs-cTnTpost 1981 (1664–2453), *p* < 0.001), as well as in CB group (hs-cTnTpre 8 (IQR 5–18 ng/L vs. hs-cTnTpost 898 (630–1206), *p* < 0.001) (Figure 2). When compared before and after ablation, patients in the PFA group had greater increases in hs-cTnT values compared with the CB group (*p* = 0.007) (Figure 2).

## 4. Discussion

In this study, we compare the effects of two ablation catheters for PVI that use different energy sources, the CB ablation and the PFA on endothelial function. Our findings indicate that PFA may exert an endothelial-sparing effect relative to cryoablation, as assessed by changes in serum vWF levels.

One potential advantage of the novel approaches to cardiac ablation based on cell electroporation is tissue specificity [9,10]. Endothelial cells are susceptible to injury, and anticoagulative properties are lost when endothelial continuity is disrupted. Our findings are consistent with emerging evidence that PFA exerts less endothelial stress compared with thermal ablation modalities. In a recent study by Osmancik et al., PFA produced markedly higher myocardial injury than radiofrequency (RF) ablation, yet levels of platelet activation and coagulation markers, including vWF antigen, fibrin monomers, and D-dimers, were not increased, and the inflammatory response was attenuated [11]. Our findings differ from those of Osmancik et al., who reported that vWF antigen and activity increased 24 h after both RF and PFA [11]. This discrepancy likely reflects the timing of biomarker assessment, as our measurements were performed immediately before and immediately after ablation, thereby capturing the acute peri-procedural dynamics of endothelial activation. Conversely, recent studies demonstrated that both RF and CB ablation significantly enhanced endothelial injury and coagulation activation [12,13]. Importantly, Koruth et al. provided preclinical proof that PFA selectively ablates myocardial tissue while preserving endothelial and vascular structures, thereby reducing the risk of thrombus formation [14]. Our findings of reduced vWF release after PFA are in line with the growing body of evidence suggesting that this modality produces myocardial lesions primarily through nonthermal electroporation, with minimal collateral injury to adjacent structures. Recent experimental work supports this concept by combining numerical modeling of tissue temperature distribution with in vivo temperature-sensitive MRI (T1-weighted imaging). These studies demonstrated only minimal heating around the catheter tip, further mitigated by blood flow, and no thermally induced lesions on MRI, confirming that the mechanism of injury during PFA is predominantly nonthermal [15]. Consistently, Yavin et al. described well-demarcated myocardial lesions characterized by edema, mononuclear lymphocytic infiltration, and arteriolar edematous thickening without evidence of coagulative necrosis or thrombus formation, further supporting a nonthermal mechanism of injury [16]. In line with these findings, the stable post-procedural vWF concentrations observed in our study suggest preserved endothelial integrity, indicating that PFA induces selective electroporation without causing significant endothelial disruption or thrombogenic surface exposure. In addition to preserving endothelial integrity, PFA appears promising in terms of neurological safety [17,18]. Our findings demonstrate an attenuated endothelial response after PFA may have potential implications for periprocedural and postprocedural anticoagulation management. Mohanty et al. recently proposed that the absence of endothelial injury after PFA could justify earlier discontinuation of oral anticoagulation in selected patients [19]. Although our data supports the concept of reduced endothelial perturbation with PFA, they do not indicate complete endothelial preservation. Therefore, these results should not be interpreted as evidence to modify current anticoagulation strategies. Rather, they highlight the need for prospective studies integrating biomarker, imaging, and clinical outcome data to determine whether the attenuated endothelial response observed with PFA translates into a lower thromboembolic risk and allows for safe adjustment of anticoagulant therapy. However, patients undergoing longer procedures with greater cumulative energy delivery are more likely to experience thromboembolic complications. Beyond the biological mechanisms, procedural aspects may also contribute, as PFA procedures were significantly shorter and associated with reduced left atrial dwell time compared with CB, thereby minimizing endothelial trauma. Although circulating vWF levels decreased after PFA compared with baseline, this should not be interpreted as a protective effect beyond catheter manipulation. Still, rather as a reflection of the endothelial-sparing properties of non-thermal electroporation compared with thermal ablation modalities. Taken together with our biomarker findings of endothelial activation, these results suggest that PFA may not only preserve vascular integrity but also lower thromboembolic and cerebral embolic risks through both biological and procedural mechanisms. One should acknowledge that endothelial cells may also undergo electroporation given their direct exposure to the electric field; nevertheless, our findings indicate that the overall acute endothelial response, reflected by vWF levels, appears attenuated. However, further large-scale and long-term studies are required to definitively confirm these potential safety advantages.

Myocardium has a lower threshold field strength to promote tissue necrosis with PFA than other tissues such as blood vessels or nerve fibers [6]. High-sensitivity cardiac troponin levels were elevated following both ablation procedures, indicating device-induced atrial myocardial injury. The higher levels could also be attributed to the larger contact surface between the PFA catheter and the endocardial tissue compared to the CB catheter. According to two previous studies, PFA elicited greater troponin release and resulted in larger antral lesions due to the flower shape of the Farawave catheter [20,21]. Alternatively, the more significant increase in cardiac troponin may indicate complete ablation inside the pulmonary vein antrum by the Farawave catheter basket shape compared to linear, antral ablation by thermal energies. It was shown that the PFA cohort’s isolation area was also more extensive than that of the CB cohort, and this could be due to the influence of the electrical field, which does not require tissue contact, or to the fact that the PFA multispline catheter can significantly extend the level of isolation in flower pose [22].

Our study has several limitations worth mentioning. First, the sample size was relatively small, which may limit the generalizability of our findings and highlights the need for confirmation in larger, prospective studies. Our study’s limitation was that we only examined laboratory biomarkers reflecting endothelial injury, which may only be a surrogate for endothelial damage, and the data only reflect the two types of ablation catheters used in the study. A limitation of our study is that endothelial injury was assessed exclusively by von Willebrand factor antigen, without measurement of other biomarkers. Future studies using a broader panel of endothelial and coagulation markers in larger cohorts are warranted to provide a more comprehensive characterization of the vascular response to different ablation modalities. In the cryoablation group, the absence of a statistically significant change in vWF may be explained by the small sample size and heterogeneity in individual biomarker trajectories. However, this study could not evaluate the efficacy or rate of complications in the two procedures. Because the study group size was small, clinical conclusions are limited; however, there may be differences between patients in the two groups (the small sample sizes may mean that important differences between the groups are missed by type 2 errors). We feel that this study provides an important conceptual framework for future research efforts. More extensive studies will be required to compare the clinical outcomes of various ablation methods and energy delivery systems.

## 5. Conclusions

In conclusion, in this prospective study comparing PFA and CB ablation for PVI, we demonstrated that PFA is associated with significantly less endothelial injury, as reflected by a reduction in circulating von Willebrand factor levels, whereas CB ablation induced a pro-coagulant endothelial response. Importantly, PFA markedly shortened left atrial dwell and ablation times, which may further contribute to a reduced thrombogenic risk. Taken together, our results suggest that PFA combines endothelial preservation with procedural efficiency, thereby potentially lowering thromboembolic complications compared with thermal ablation modalities. While these findings reinforce the concept of PFA as an “endothelial-sparing” ablation modality, additional large-scale and long-term studies are needed to confirm the vascular and neurological safety profile of this novel energy source.

## Figures and Tables

**Table 1:** The baseline, clinical, echocardiographic, and laboratory data of enrolled patients.

Variable	Pulsed-Field Ablation n = 14	Cryoballoon Ablation n = 11	*p*
Age	61.79 ± 10	60.09 ± 8	0.677
Female sex	9 (64.3%)	5 (45.5%)	0.346
Body mass index, (kg/m^2^)	30 ± 5	27 ± 3	0.175
Arterial hypertension	7 (50%)	6 (54.5%)	0.821
Smoking	5 (36%)	4 (36%)	0.973
Systolic blood pressure (mmHg)	116 ± 13	120 ± 11	0.450
Duration of AF since first documented episode (months)	124.3 ± 95.6	81.2 ± 83.9	0.250
RAAS inhibitors	5 (36%)	3 (27%)	0.653
Beta-blockers	9 (64%)	6 (54%)	0.622
Statins	6 (42%)	6 (54%)	0.561
Left atrium diameter (mm)	41 ± 3.6	42 ± 7	0.415
Left ventricular ejection fraction (%)	62.8 ± 3.4	65.3 ± 6.7	0.240
CHA_2_DS_2_-VASc score	1.79 ± 1.31	1.45 ± 1.36	0.545
NT-proBNP (pg/mL)	300 ± 295	323 ± 303	0.848
vWF levels baseline (%)	129.5 ± 32.1	135.4 ± 49.8	0.726
hs-troponin T baseline (ng/L)	8.8 ± 6.0	10.3 ± 6.3	0.551
D-dimer baseline (mg/L)	0.34 ± 0.26	0.35 ± 0.45	0.937
Platelets level baseline (×10^3^)	256.5 ± 54.24	246.4 ± 49.82	0.638
eGFR baseline	76.14 ± 13.52	74.7 ± 23.19	0.850
White blood cells (10^3^ × cells/lL) baseline	7.5 ± 0.9	7.3 ± 1.8	0.854
Hemoglobin (g/L)	142.2 ± 15.5	144.3 ± 11.6	0.718
Fasting glucose (g/dL)	5.8 ± 1.3	5.8 ± 0.7	0.921
Total cholesterol (mmol/L)	5.5 ± 1.9	4.4 ± 1.3	0.125

Continuous variables are expressed as mean ± SD. Categorical variables are indicated as numbers (percentages) unless otherwise stated. n: number; SD: standard deviation; CHA2DS2-VASC score: congestive heart failure, hypertension, age ≥ 75 years, diabetes mellitus, stroke/transient ischemic attack/thromboembolism, vascular disease (prior myocardial infarction, peripheral vascular disease, or aortic atherosclerosis), age (65–74 years), sex category (female); vWF: Von Willebrand factor; RAAS: renin–angiotensin–aldosterone system.

**Table 2:** Procedural characteristics of the study population.

Procedural Characteristics	Pulsed-Field Ablation n = 14	Cryoballoon Ablation n = 11	*p*
Procedure time (min.)	59.3 ± 25.4	94.1 ± 28.4	0.005
Fluoroscopy time (min.)	12.8 ± 7.4	12 ± 7	0.888
LA dwell time (min.)	44.3 ± 14.8	79.3 ± 21.9	<0.001
Total heparin dose (IU)	18,000 IU (IQR 13,500–20,000)	20,000 (IQR 13,000–23,000)	0.181

Continuous variables are expressed as mean ± SD or median (interquartile range-IQR); n: number; SD: standard deviation.

## Data Availability

The data presented in this study are available on request from the corresponding author. The data are not publicly available because some of the data set will be used for further research.
